# Food Nitrogen Footprint of the Indian Subcontinent Toward 2050

**DOI:** 10.3389/fnut.2022.899431

**Published:** 2022-05-20

**Authors:** Aurup Ratan Dhar, Azusa Oita, Kazuyo Matsubae

**Affiliations:** ^1^Department of Environmental Studies for Advanced Society, Graduate School of Environmental Studies, Tohoku University, Sendai, Japan; ^2^Institute for Agro-Environmental Sciences, National Agriculture and Food Research Organization, Tsukuba, Japan

**Keywords:** EAT-Lancet planetary health diet, culture and religion, food consumption, nitrogen use efficiency, religion-sensitive footprint method

## Abstract

Substantial loss of nitrogen (N) in reactive forms (nitrogen species except for N_2_) induced by agro–food system is a cause of the environmental degradation and harms human health. The main factors influencing the food N footprint of the Indian Subcontinent (ISC) are the nitrogen use efficiency (NUE) of crop cultivation and religious dietary cultures. In this study, we assess the food N footprint of the ISC and establish reduction scenarios toward 2050. We used a religion-sensitive N-Calculator method and food consumption data from the Food and Agriculture Organization of the United Nations to estimate the food N footprint of the ISC of different religious communities. We developed four reduction scenarios as follows: The business-as-usual scenario; a 30% increase in the crop cultivation NUE; altered protein supplies to the healthy EAT–Lancet reference diet considering religious food taboos; and an integrated approach with a 30% increase in the NUE increase and the altered diet. We used the long short-term memory recurrent neural network approach to predict the future. The study revealed that the average food N footprint per-capita per-year increased from 7.94 kg-N in the 1960s to 8.43 kg-N in the early 2010s, and the crop cultivation NUE was reduced to less than 40%. Buddhists had the lowest footprint over the period. An increase in the NUE of the crop cultivation and an altered diet results in a 13% reduction in the N footprint compared to the business-as-usual scenario. We conclude that improved crop cultivation NUEs and an altered religion-specific healthy diet would reduce the N footprint.

## Introduction

Nitrogen (N) is one of the essential key elements for all living organisms. Reactive forms of N (N species other than N gas), denoted by Nr, are reasonably scarce, often limiting plant growth in ecosystems. However, the widespread overuse of N fertilizers for food production results in excessive Nr in the environment, causing soil, air, and water pollution. Excessive Nr is associated with higher greenhouse gas (GHG) emissions and eutrophication of the waterways. The N pollutants in the air released from fuel combustion are also known to be a factor in human respiratory diseases (1).

The N footprint of food quantifies Nr loss to the environment from both food production and food consumption (2). Most of the Nr loss takes place during food production and some loss occurs during food consumption. The food N footprint of a country is influenced by a number of behavioral, technical, and socioeconomic factors, including food choice, food waste, manure N recycling rate, the nitrogen use efficiency (NUE) of food production (i.e., how much of the N input is still in the food eaten), wastewater treatment, population growth, gender, and age difference (3). Among these factors, the decrease in the NUE of food production is a major concern in food Nr loss (1). Globally, the NUE of crop cultivation is low, at close to 47% on average over the last three decades (4). Improving the NUE of crop production (on farms) is essential for effective N management in cropping systems. At the consumption level, food choice and nutrition depend on multiple cultural and religious food directives (5). The increased preference for animal protein has escalated Nr loss to the environment, particularly in China (6). The Indian Subcontinent (ISC) is comprised of Bangladesh, India, Pakistan, Sri Lanka, Nepal, and Bhutan, as shown in Supplementary Figure 1 and is the world's most religiously diverse region with 36% of the population Muslim, 35% Hindu, 26% Buddhist, 2% Christian, and 1% followers of other religions (7). Based on a case study of India, Dhar et al. (3) pointed out the importance of increasing the NUE in food production and choosing a diet with a lower N footprint aligned with religious-based dietary regulations to reduce the N footprint of food. In addition to choosing food items associated with less Nr loss to the environment, maintaining a balanced, nutritional diet is also important. At the time of writing this article, there was no research found in the literature that focused on all of the following important aspects of food consumption: the N footprint, religion-based dietary regulations, and nutrition.

In this study, we considered the N footprint of the ISC in the years approaching the 2050s with nutrition and religion-based dietary regulations. The four scenarios assessed were the business-as-usual scenario (BAU scenario), a scenario with an increase in the NUE of crop cultivation (NUE scenario), a scenario with people in each religious community following a healthy diet (EAT–Lancet scenario), and a combination of the NUE and the EAT–Lancet approaches (integrated scenario). We expect that the results of this study will help people choose a religion-specific healthy diet with a low N footprint and support the policymakers in their efforts on formulating policy measures for effective and efficient N management in food systems.

## Methods

### Food Nitrogen Footprint

The food N footprint of the ISC was calculated by applying the religion-sensitive N-Calculator method developed by Dhar et al. (3). The religion-specific dietary laws and rules of each of the four major religions were considered when estimating the consumption of each food item by their followers. Since the Hindu faith confines the diet to plant-based and dairy products, Hindus were regarded as lacto–vegetarians. Buddhists were characterized as vegetarians because Buddhism allows only plant-based products to be eaten. Since there are no category-wide food restrictions, Muslims and Christians were classified as non-vegetarians. The food items on the food balance sheets (FBSs) provided by the Food and Agriculture Organization (FAO) were categorized according to the N intake by each religious community. The FBSs and the fertilizer data by FAO from 1961 to 2013 were used as the time-series data of the maximum available time range (8).

The per-capita food N footprint (food NF) of the ISC was calculated as an average of the per-capita food NF of the countries in the ISC. The food NF of country, *c*, in the ISC was estimated as the sum of food production N footprint (food production NF) and food consumption N footprint (food consumption NF) as follows (Eq. 1):


(1) $$\begin{array}{l} \text{Food }{\text{NF}}_{c}\;=\;\sum_{m=1}^{y}\sum_{n=1}^{x}W_{nc}(\text{Food production }{\text{NF}}_{mnc}\\ +\text{ Food consumption }{\text{NF}}_{mnc}) \end{array}$$

where *x* (=5) is the number of religious communities, *y* (=94) is the total number of food items, *m* is a particular food item, *n* is a particular religious group, and *W*_*nc*_ is the population ratio of religious group *n* in the country, *c*.

The per-capita food production NF and food consumption NF of country *c* in the ISC were calculated as follows (Eqs 2, 3):


(2) $$\begin{array}{l} \text{Food production }{\text{NF}}_{mnc}\;=\text{ Per - capita protein }{\text{supply}}_{mnc}\\ \times \text{ N content of supplied }{\text{protein}}_{m}\;\times \;\left(1\;-\text{ Food }{\text{waste}}_{mc}\right)\\ \times \text{ Trade - considered }{\text{VNF}}_{mc} \end{array}$$

(3) $$\begin{array}{l} \text{Food consumption }{\text{NF}}_{mnc}\;=\text{ Per - capita protein }{\text{supply}}_{mnc}\\ \times \text{ N content of supplied }{\text{protein}}_{m}\;\times \;\left(1\;-\text{ Food }{\text{waste}}_{mc}\right)\\ \times \;(1\;-\text{ Denitrification ratio}) \end{array}$$

The ratio of denitrification is set to 0% because there is no evidence regarding sewage treatment with N removal technology in the ISC.

Thus, the food N footprint is largely affected by the following two factors: The amount and composition of food protein consumption and the NUE of crop cultivation for food and feed.

### Virtual Nitrogen Factors

The virtual N factors (i.e., VNFs) indicate the amount of Nr lost to the environment during the food production process and are not contained in the consumed food. The VNFs are calculated by aggregating N use efficiencies (i.e., NUEs) and the ratios of each food production process (9). On the basis of food self-sufficiency ratios (8), the trade-considered VNFs of country, *c*, (Table 1) were computed as follows (Eq. 4):


(4) $$\begin{array}{l} \text{Trade - considered }{\text{VNF}}_{mc}\;=\text{ Self - sufficiency }{\text{ratio}}_{mc}\\ \times \text{ Domestic }{\text{VNF}}_{mc}\;+\;\left(1\;-\text{ Self - sufficiency }{\text{ratio}}_{mc}\right)\\ \times \text{ Average domestic VNF of six countries in the }{\text{ISC}}_{m} \end{array}$$

**Table 1:** Trade-considered VNFs of the ISC.

**Aggregated food categories**	**VNF by decades (kg-N loss kg-N**^**−1**^ **intake)**					
	**1960s**	**1970s**	**1980s**	**1990s**	**2000s**	**2010s (up to 2013)**
**Bangladesh**						
Cereals	1.60	1.78	1.91	2.32	2.15	1.94
Starchy roots	1.73	1.78	2.16	2.91	2.55	2.03
Oil crops and pulses	2.34	2.50	2.88	3.25	3.25	3.05
Vegetables	1.91	2.34	3.23	4.63	4.86	4.11
Fruits	2.20	2.66	3.66	6.55	8.09	7.80
Other plant products	1.91	2.33	3.19	4.56	4.83	3.99
Meat and offal	4.06	4.03	4.02	4.07	4.04	3.92
Milk and dairy products	12.16	10.15	9.35	9.66	6.68	6.16
Eggs	4.82	4.36	3.37	3.14	3.16	2.98
Fish and seafood	1.27	1.28	1.33	1.37	1.40	1.51
**India**						
Cereals	1.90	1.91	2.06	2.23	2.41	2.43
Starchy roots	1.45	1.34	1.36	1.45	1.51	1.47
Oil crops and pulses	2.54	2.53	2.71	2.53	2.64	2.42
Vegetables	1.63	1.74	1.98	2.22	2.40	2.43
Fruits	2.25	2.46	2.78	3.12	3.67	3.81
Other plant products	1.78	1.88	2.11	2.34	2.46	2.45
Meat and offal	4.13	4.16	4.17	4.09	4.09	3.90
Milk and dairy products	12.12	10.12	9.32	9.58	6.90	6.48
Eggs	4.88	4.78	3.76	3.09	3.46	3.51
Fish and seafood	1.35	1.42	1.60	1.71	1.64	1.57
**Pakistan**						
Cereals	1.42	1.21	1.18	1.41	1.48	1.51
Starchy roots	1.11	1.08	1.27	1.49	1.62	1.61
Oil crops and pulses	3.11	2.95	3.33	3.55	3.68	3.54
Vegetables	1.69	1.61	1.88	2.43	3.20	3.56
Fruits	3.74	2.08	2.82	3.80	5.86	7.25
Other plant products	1.71	1.66	1.98	2.54	3.11	3.28
Meat and offal	4.20	4.15	4.04	3.99	3.98	3.81
Milk and dairy products	12.00	9.92	9.04	9.24	6.51	5.99
Eggs	4.78	4.07	3.00	2.69	2.68	2.53
Fish and seafood	1.38	1.42	1.73	1.79	1.56	1.51
**Sri Lanka**						
Cereals	2.02	2.04	2.12	2.34	2.55	2.23
Starchy roots	3.03	3.50	2.60	3.07	3.25	2.50
Oil crops and pulses	3.92	4.29	4.64	5.15	5.25	4.27
Vegetables	2.34	2.72	3.24	3.40	3.58	3.18
Fruits	4.02	2.70	3.27	6.09	8.89	8.60
Other plant products	2.32	1.37	3.20	0.62	3.53	3.12
Meat and offal	4.02	3.92	3.84	3.66	3.71	3.52
Milk and dairy products	12.18	10.16	9.34	9.60	6.81	6.30
Eggs	5.01	4.80	3.82	3.14	3.76	3.53
Fish and seafood	1.30	1.36	1.39	1.41	1.38	1.46
**Nepal**						
Cereals	1.31	1.42	1.55	1.61	1.53	1.61
Starchy roots	1.56	1.68	1.81	1.66	1.49	1.48
Oil crops and pulses	3.00	2.90	2.92	2.73	2.37	2.29
Vegetables	1.43	1.58	1.67	1.73	1.64	1.52
Fruits	1.00	1.00	1.83	5.07	5.67	5.93
Other plant products	1.44	1.60	1.76	1.78	1.78	1.76
Meat and offal	4.05	4.10	4.20	4.23	4.30	4.27
Milk and dairy products	11.93	9.93	9.16	9.37	6.66	6.28
Eggs	4.62	4.15	3.90	5.45	3.64	3.19
Fish and seafood	1.25	1.25	1.25	1.25	1.26	1.29
**Bhutan**						
Cereals	1.43	1.56	1.86	2.11	2.05	1.59
Starchy roots	1.64	1.70	1.71	1.84	2.03	1.84
Oil crops and pulses	3.26	3.00	2.62	3.08	1.96	2.24
Vegetables	2.13	2.16	1.99	2.47	2.20	2.49
Fruits	3.67	3.83	3.75	3.57	4.52	4.26
Other plant products	2.12	2.15	2.04	2.48	2.27	2.54
Meat and offal	4.07	4.12	4.22	4.26	4.30	4.18
Milk and dairy products	12.04	10.01	9.24	9.48	6.67	6.18
Eggs	4.71	4.21	3.96	5.51	3.61	2.95
Fish and seafood	1.25	1.25	1.25	1.25	1.26	1.29
**ISC average**						
Cereals	1.61	1.65	1.78	2.00	2.03	1.89
Starchy roots	1.75	1.85	1.82	2.07	2.08	1.82
Oil crops and pulses	3.03	3.03	3.18	3.38	3.19	2.97
Vegetables	1.86	2.02	2.33	2.81	2.98	2.88
Fruits	2.81	2.45	3.02	4.70	6.12	6.27
Other plant products	1.88	1.83	2.38	2.39	3.00	2.85
Meat and offal	4.09	4.08	4.08	4.05	4.07	3.93
Milk and dairy products	12.07	10.05	9.24	9.49	6.70	6.23
Eggs	4.80	4.40	3.64	3.83	3.38	3.12
Fish and seafood	1.30	1.33	1.42	1.46	1.42	1.44

### Nitrogen Use Efficiency

When calculating the trade-considered VNFs, the cultivation NUE of crop, *b*, produced in the country, *c*, was computed as follows (Eq. 5):


(5) $$\begin{array}{l} {\text{NUE}}_{bc}\\ =\;\sum_{b=1}^{h}\frac{N_{{\text{cont}}_{bc}}}{N_{{\text{fert}}_{bc}}\;+\;N_{{\text{man}}_{bc}}\;+\;N_{{\text{adep}}_{bc}}\;+\;N_{{\text{bfix}}_{bc}}\;+\;N_{{\text{seed}}_{bc}}} \end{array}$$

where *h* (=82) is the total number of crops produced, *N*_cont_ is the N content in the harvested crop (10), *N*_fert_ is the N fertilizers applied (8), *N*_man_ is the livestock manure applied (1), *N*_adep_ is the atmospheric N deposition (11), *N*_bfix_ is the biological N fixation (12), and *N*_seed_ is the N content in crop seed (6).

The relationship between domestic VNF and NUE of crop cultivation is expressed as follows (Eq. 6):


(6) $$\begin{array}{l} {\text{VNF}}_{bc\;}=\;\frac{1}{(\text{Cultivation }{\text{NUE}}_{bc}\;\times \text{ Processing }{\text{NUE}}_{bc}}\\ \;\times \text{ Consumer - level utilization }{\text{NUE}}_{bc}) \end{array}$$

The additional data sources used for calculation are provided in the Supplementary Document.

### Scenario Analysis

**Figure 1:**
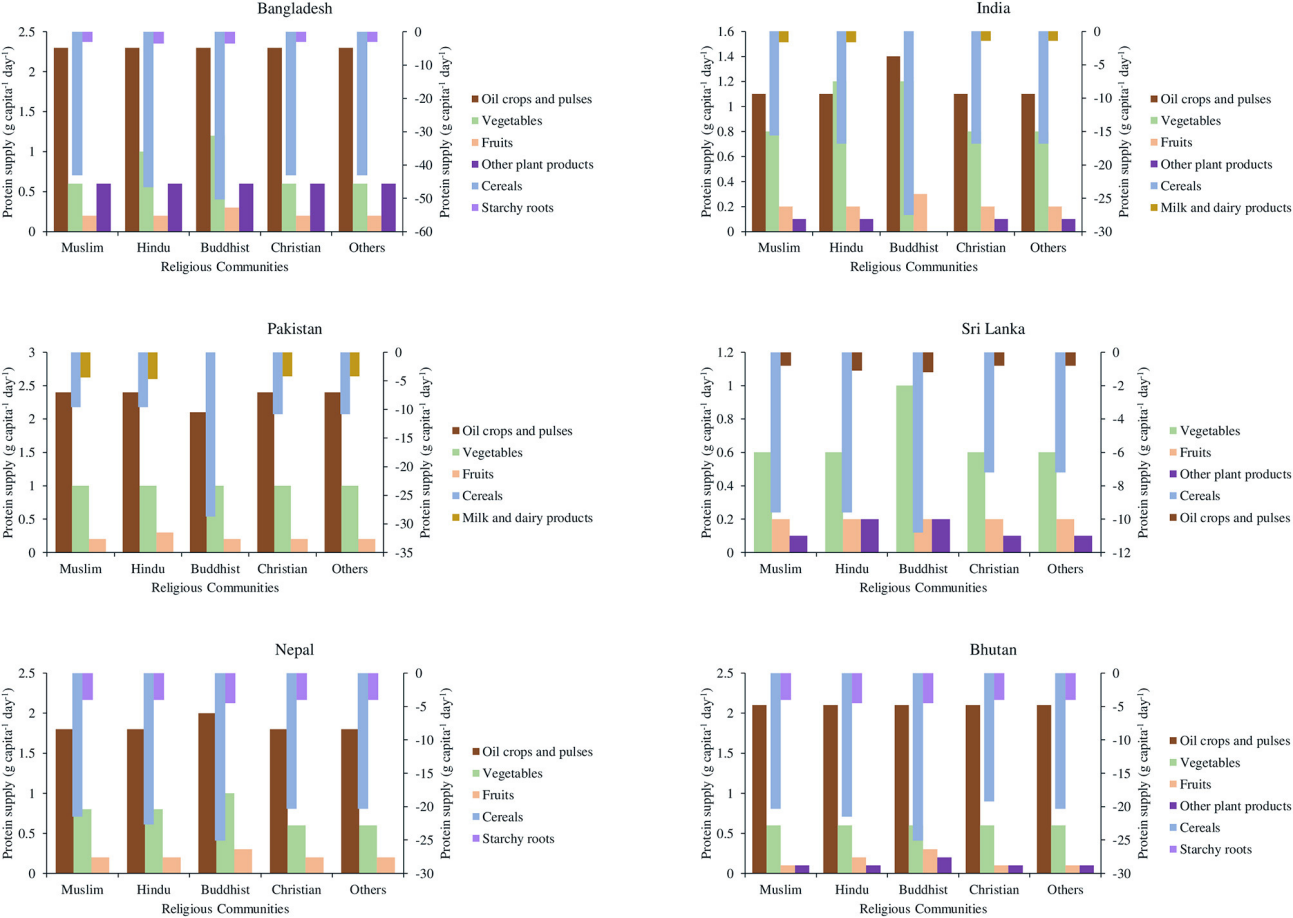
Recommended religion-sensitive alterations in protein supply relative to 2013 protein supply base. Positive values indicate increase and negative values indicate decrease in protein supply.

We have assessed the four scenarios of the food N footprint of the ISC for the years leading up to 2050. The following four scenarios were assessed: (a) The BAU scenario, in which each person in a religious group eats the same amount of each food item as in 2013 and the food is produced in the same way as it was in 2013, (b) the NUE scenario, in which the crop cultivation NUEs are increased by 30% relative to the NUEs in 2013, (c) the EAT–Lancet scenario, with the 2013 religion-sensitive protein altered for a religion-sensitive healthy diet for people and the environment, as shown in Figure 1, and (d) the integrated scenario, which is a combined approach including the NUE scenario and the EAT–Lancet scenario.

**Figure 2:**
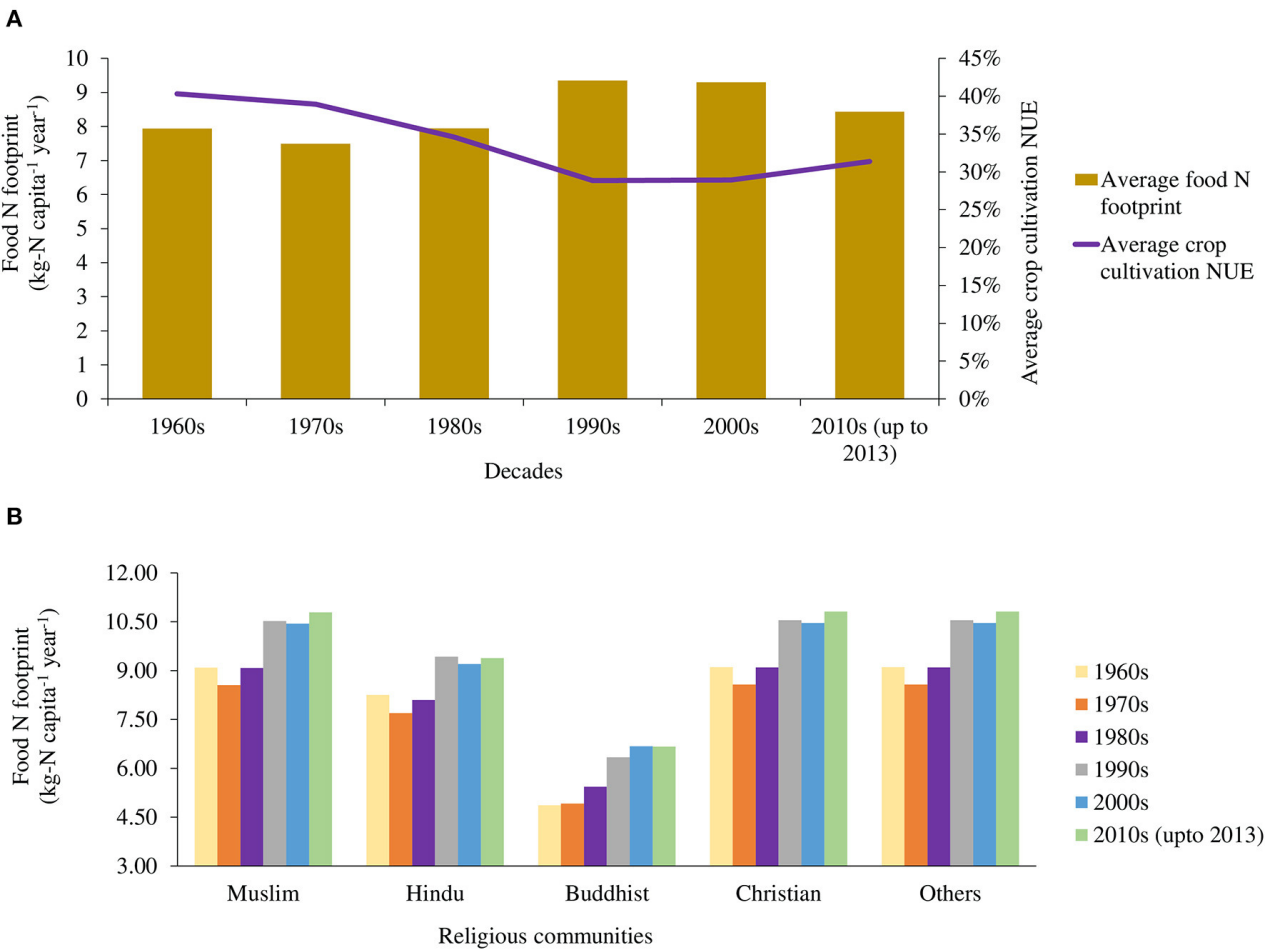
Food N footprint of the ISC **(A)** regional average with crop cultivation NUE and **(B)** by religious communities.

The scenarios were built on the global projection of food production NUE by 2050 and a planetary health diet that is healthy for both people and planet recommended by the EAT–Lancet Commission (13, 14). Based on the year of 1997, Mosier et al. (14) estimated the global NUE in food production as nearly 30%, and projected that it would further increase by 30% by 2050 from what was in 1997. Based on the steady increase in the crop cultivation NUE of the ISC over the last 20 years shown in Figure 2A, we assumed that the crop cultivation NUE in the ISC countries in the NUE scenario will be 30% more than that in the BAU scenario from 2014 to 2050. For the EAT–Lancet scenario, we incorporated the food composition in the daily diet recommended by the EAT–Lancet Commission to attain the planetary health diet, as shown in Supplementary Table 1, with a daily calorie intake of 2,500 kcal for adults (13). The United States Department of Agriculture recommends an average intake of 51-g protein per day for an adult. We proposed the religion-sensitive alterations to protein supplies as shown in Figure 1 after considering these daily nutritional recommendations in terms of protein consumption. As shown in Table 2, the share of cereals, starchy roots, and “milk and dairy products” was decreased, and the share of fruits, vegetables, “oil crops and pulses,” and other plant products was increased for daily diets based on the country-specific data from the FBSs in 2013 (8).

**Table 2:** Recommended religion-sensitive alterations in food composition (% of food share) in EAT–Lancet planetary health diet based on FBSs in 2013.

**Aggregated food categories**	**EAT–lancet diet**	**Food consumption by religious communities**									
		**Muslim**		**Hindu**		**Buddhist**		**Christian**		**Others**	
		**Actual intake**	**Recommended alteration from actual intake**	**Actual intake**	**Recommended alteration from actual intake**	**Actual intake**	**Recommended alteration from actual intake**	**Actual intake**	**Recommended alteration from actual intake**	**Actual intake**	**Recommended alteration from actual intake**
**Bangladesh**											
Cereals	32	68	−36	71	−39	74	−42	68	−36	68	−36
Starchy roots	2	8	−6	9	−7	9	−7	8	−6	8	−6
Oil crops and pulses	18	3	+15	3	+15	3	+15	3	+15	3	+15
Vegetables	3	1	+3	1	+5	1	+6	1	+3	1	+3
Fruits	5	2	+5	2	+6	2	+8	2	+5	2	+5
Other plant products	27	10	+19	10	+20	11	+20	10	+19	10	+19
Meat and offal	4	1	–	–	–	–	–	1	–	1	–
Milk and dairy products	6	4	–	4	–	–	–	4	–	4	–
Eggs	1	–	–	–	–	–	–	–	–	–	–
Fish and seafood	2	3	–	–	–	–	–	3	–	3	–
**India**											
Cereals	32	45	−13	46	−14	55	−23	46	−14	46	−14
Starchy roots	2	4	–	5	–	5	–	4	–	4	–
Oil crops and pulses	18	8	+7	8	+7	9	+9	8	+7	8	+7
Vegetables	3	3	+4	3	+6	3	+6	3	+4	3	+4
Fruits	5	5	+6	5	+6	5	+8	5	+6	5	+6
Other plant products	27	20	+3	20	+2	23	–	20	+3	20	+3
Meat and offal	4	1	–	–	–	–	–	1	–	1	–
Milk and dairy products	6	13	–	13	–	–	–	12	−6	12	−6
Eggs	1	–	–	–	–	–	–	–	–	–	–
Fish and seafood	2	1	–	–	–	–	–	1	–	1	–
**Pakistan**											
Cereals	32	40	−8	40	−8	56	−24	41	−9	41	−9
Starchy roots	2	2	–	3	–	3	–	2	–	2	–
Oil crops and pulses	18	3	+16	3	+16	4	+14	3	+16	3	+16
Vegetables	3	1	+5	1	+5	1	+5	1	+5	1	+5
Fruits	5	2	+6	2	+7	3	+5	2	+6	2	+6
Other plant products	27	24	–	25	–	33	–	24	–	24	–
Meat and offal	4	3	–	–	–	–	–	3	–	3	–
Milk and dairy products	6	25	–	26	–	–	–	24	−18	24	−18
Eggs	1	–	–	–	–	–	–	–	–	–	–
Fish and seafood	2	–	–	–	–	–	–	–	–	–	–
**Sri Lanka**											
Cereals	32	40	−8	40	−8	41	−9	38	−6	38	−6
Starchy roots	2	2	–	2	–	3	–	2	–	2	–
Oil crops and pulses	18	23	−5	25	−7	26	−8	23	−5	23	−5
Vegetables	3	1	+3	1	+3	1	+5	1	+3	1	+3
Fruits	5	2	+5	3	+6	3	+6	2	+4	2	+4
Other plant products	27	23	+5	25	+6	26	+6	25	+4	25	+4
Meat and offal	4	1	–	–	–	–	–	1	–	1	–
Milk and dairy products	6	4	–	4	–	–	–	4	–	4	–
Eggs	1	1	–	–	–	–	–	1	–	1	–
Fish and seafood	2	3	–	–	–	–	–	3	–	3	–
**Nepal**											
Cereals	32	50	−18	51	−19	53	−21	49	−17	49	−17
Starchy roots	2	10	−8	10	−8	11	−9	10	−8	10	−8
Oil crops and pulses	18	4	+12	4	+12	4	+13	4	+12	4	+12
Vegetables	3	3	+3	3	+4	4	+5	3	+3	3	+3
Fruits	5	4	+6	4	+6	5	+7	4	+5	4	+5
Other plant products	27	21	–	22	–	23	–	22	–	22	–
Meat and offal	4	2	–	–	–	–	–	2	–	2	–
Milk and dairy products	6	6	–	6	–	–	–	6	–	6	–
Eggs	1	–	–	–	–	–	–	–	–	–	–
Fish and seafood	2	–	–	–	–	–	–	–	–	–	–
**Bhutan**											
Cereals	32	49	−17	50	−18	53	−21	48	−16	49	−17
Starchy roots	2	10	−8	11	−9	11	−9	10	−8	10	−8
Oil crops and pulses	18	4	+14	4	+14	4	+14	4	+14	4	+14
Vegetables	3	3	+3	3	+3	4	+3	4	+3	3	+3
Fruits	5	4	+3	4	+6	5	+7	4	+3	4	+3
Other plant products	27	22	+5	22	+4	23	+6	22	+4	22	+5
Meat and offal	4	2	–	–	–	–	–	2	–	2	–
Milk and dairy products	6	6	–	6	–	–	–	6	–	6	–
Eggs	1	–	–	–	–	–	–	–	–	–	–
Fish and seafood	2	–	–	–	–	–	–	–	–	–	–

*Source: Hackett et al. (7), Willett et al. (13), and FAO (15)*.

To estimate the approximate values of the food N footprint from 2014 to 2050, we applied the long short-term memory recurrent neural network (i.e., LSTM–RNN) approach of the machine learning method (16). This method incorporates a class of loop networks for processing data, with the output depending on the previously computed values. The food N footprint values for each year were estimated in what is known as a “neural network cell,” as follows (Eq. 7):


(7) $$\begin{array}{r} h_{t}=o_{t}\text{tanh}\left(C_{t}\right) \end{array}$$

where *h*_*t*_ is the current output at time *t, o*_*t*_ is the output gate used for computing the output values, and *C*_*t*_ is the cell states at time *t*.

## Results

### Historical Food Nitrogen Footprint

#### Food Nitrogen Footprint of the ISC and the ISC Countries

**Figure 3:**
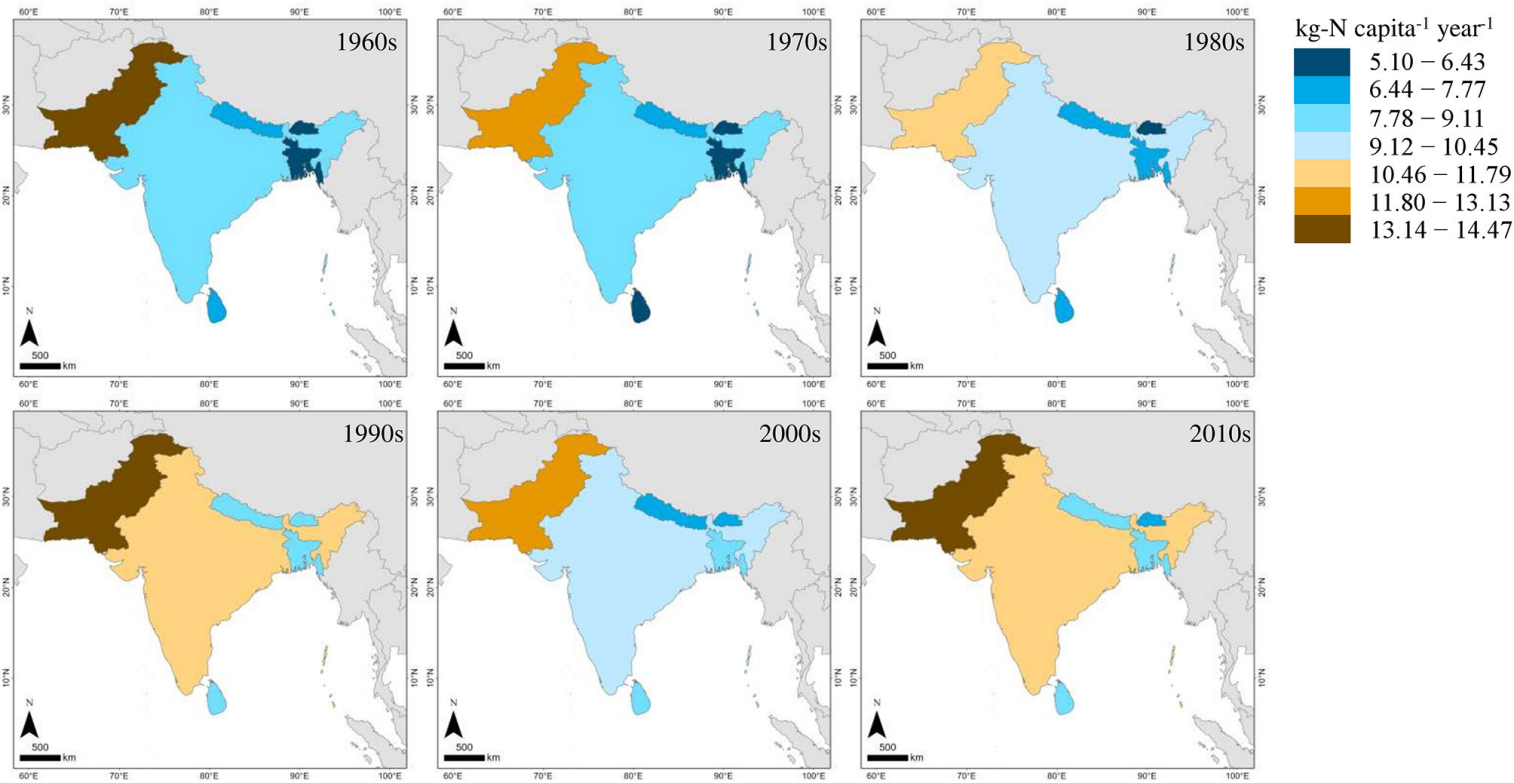
Observed food N footprint of the countries in the ISC from the 1960s to the 2010s.

The per-capita annual food N footprint of the ISC has increased slightly over the last six decades, from 7.94 in the 1960s to 8.43 kg-N in the early 2010s, with a decline in the crop cultivation NUE in this period (Figure 2A). Although the crop cultivation NUE increased slightly in the recent years, it was 40% or lower throughout the period (Supplementary Figure 2). As the VNFs are inversely proportional to the NUEs, the slight decrease in the average VNFs for most of the food items was accompanied by an increase in the crop cultivation NUE since the turn of the century. The VNFs of the animal-based foods were higher than that of the plant-based foods at all the times in the ISC region. The high 12.07 VNF of “milk and dairy products” in the 1960s decreased considerably to 6.23 in the decade beginning in 2010, and the lowest VNF was consistently that of “fish and seafood” (Table 1). In terms of country-specific per-capita annual food N footprints, the highest footprint in the early 2010s was observed in Pakistan (13.60 kg-N) while the lowest one was for Bhutan (7.34 kg-N; Figure 3). The per-capita annual food N footprints of Bangladesh, India, Sri Lanka, and Nepal were found to be 8.34 kg-N, 11.02 kg-N, 8.50 kg-N, and 8.36 kg-N, respectively.

#### Religion-Specific Food Nitrogen Footprint

**Figure 4:**
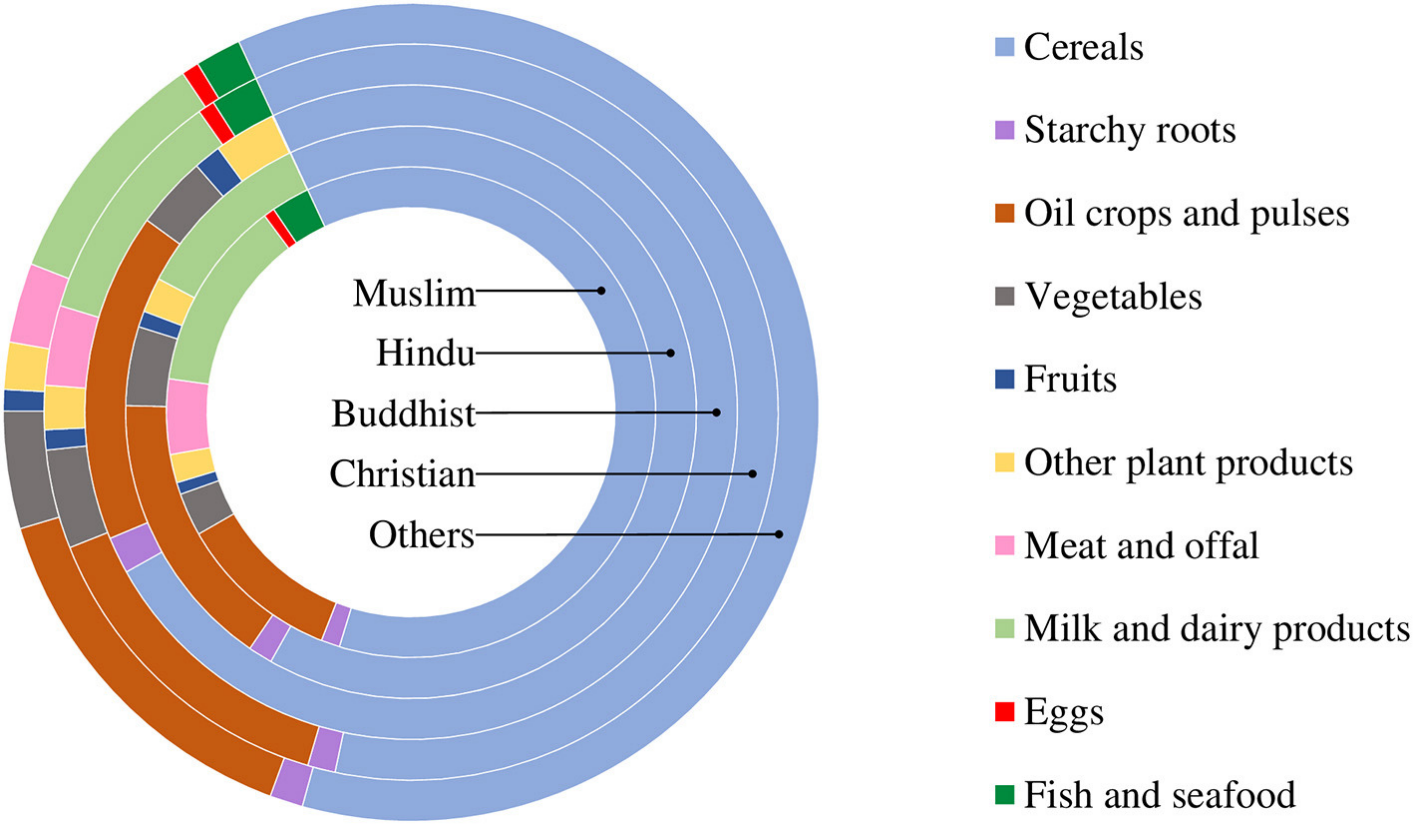
N consumed from food by religious communities in the ISC.

Food consumption patterns differed among the religious groups (Figure 4). The food N footprints of all religious communities had increasing trends over the time. Among the religious communities, the lowest and highest food N footprint were for Buddhists and Christians, respectively. The per-capita annual food N footprints in the early 2010s for the religious communities were estimated at 10.79 kg-N for Muslims, 9.39 kg-N for Hindus, 6.66 kg-N for Buddhists, and 10.81 kg-N for Christians (Figure 2B). Among the food items, the consumed N was the highest from cereals (more than 60% for all religious communities), followed by “oil crops and pulses” and “milk and dairy products” (Figure 4).

### Expected Food Nitrogen Footprint by 2050

**Figure 5:**
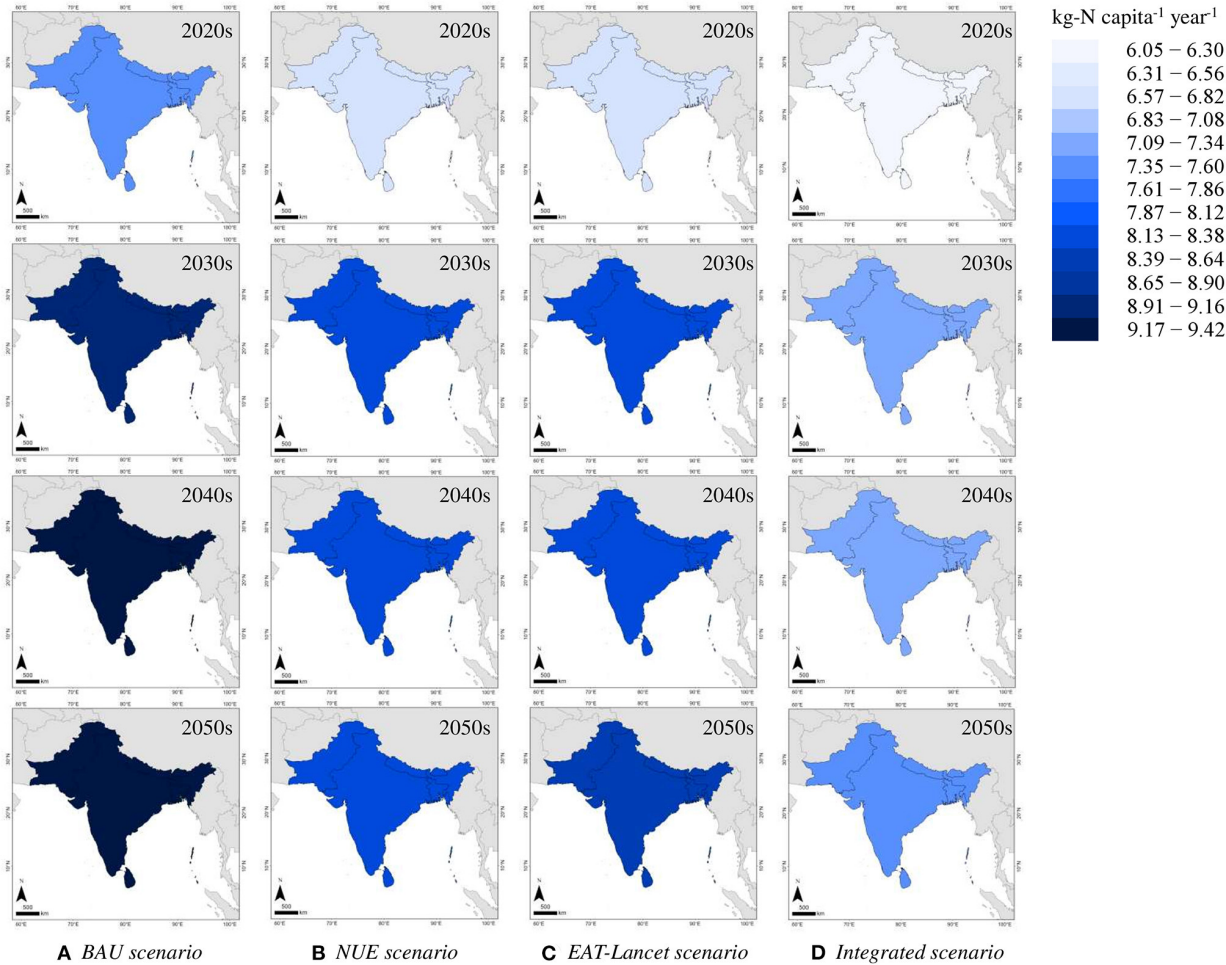
Expected food N footprint of the ISC from the 2020s to the 2050s under different scenarios **(A)** BAU scenario, **(B)** NUE scenario, **(C)** EAT-Lancet scenario, and **(D)** Integrated scenario.

By 2050, the expected per-capita annual food N footprint of the ISC is expected to be nearly 10% higher than the average during the decade from 2010 to 2050, according to the BAU scenario (9.27 kg-N). However, it is about 3% lower using the NUE scenario (8.14 kg-N), 1% using the EAT–Lancet scenario (8.34 kg-N), and 13% lower using the Integrated scenario (7.31 kg-N; Figure 5). Assuming that the values will continue to decline as they have since the early 2000s, the food N footprint is expected to decrease until the mid-2020s, and then increase gradually in the years leading to 2050. The root mean square error (RMSE) values for the BAU scenario, the NUE scenario, the EAT–Lancet scenario, and the integrated scenario were computed to be 0.32, 0.22, 0.33, and 0.20, respectively (Supplementary Figure 3).

## Discussion

### Contribution of Nitrogen Use Efficiency in Food Nitrogen Footprint

The food N footprint of the ISC is subject to religious faiths and is culturally sensitive (Figure 2B). However, the NUEs of food items are not strongly affected by the cultural and religious restrictions on food production, since all four religions accept crop cultivation and rearing livestock as domestic animals as a means of making a living (3). The NUEs of specific food items are significant because consuming food items with lower NUEs leads to higher food N footprint, as shown in Figure 2A. On the other hand, food consumption is largely affected by religious directives, as shown in Figure 4.

Improving the NUEs of crop cultivation not only increases crop productivity but also is an effective means of reducing environmental degradation. Among the average NUEs of crop cultivation for the ISC countries, the lowest was Sri Lanka, which was attributed to its low soil N content, inefficient irrigation system, overfertilization, improper use of other production inputs, and low N recycling and recovery rate (17). The average NUE of Pakistan was notably higher than all other countries in the ISC in the 1960s−1980s although it dramatically decreased in the later decades mainly due to the misuse of fertilizers. Pakistan had a high NUE in the 1960s−1980s – thanks to its high yield crop varieties, even with soil N deficiency. However, the increased N application in the following years since then resulted in a reduction in the crop yield (18). The average NUE of Bangladesh, Nepal, and Bhutan gradually increased after the late 1990s despite a rapid decline during the 1960s−1990s, whereas that of India did not increase much in the 2000s with the stabilized consumption of N fertilizers (19).

### Comparing Findings With Other Regions of the World

The food N footprint of different regions around the world is based on remarkably different food consumption behaviors of the people. The food N footprint of the ISC is in line with the low Indian food N footprint due to India's high dependance on plant-based foods, as regulated by both culture and the dominant religions in India (3, 9). The difference between non-vegetarians, Muslims and Christians, and vegetarians, Hindus and Buddhists, could be seen in their consumption of N, from “meat and offal,” eggs, and “fish and seafood” or none of those. In China, the major contributor to the food N footprint was also cereals, whereas in Japan, it was found to be highly dependent on “meat and offal,” representing 37% of the food N footprint (9). Similarly, the food N footprints of North America and Australia were dominated by animal-based foods, particularly red meat (2, 20, 21), whereas dairy products were dominant in the European Union (22). Sub-Saharan Africa (SSA) had the lowest food N footprint among all the investigated regions of the world, reflecting its low protein consumption (23).

### Toward Reducing Nitrogen Footprint of Food

While a shift to a low-protein healthy diet in the coming decades will help to reduce the food N footprint, N deficiencies from food intake result in food insecurity and malnutrition, as evident in SSA (24). A healthy diet for people and the environment is necessary to safeguard the daily nutritional requirements without causing environmental degradation. We estimated the current average per-capita daily calorie intake in the ISC to be 2,245 kcal in 2013, similar to the 2,192 kcal for India, but moderately lower than the world average of 2,901 kcal (8, 25). As revealed by the scenario assessment (Figure 5), the food N footprint of the ISC is expected to increase from 2010 to 2050 for the BAU scenario, but it decreased by 13% in the integrated scenario because of the enhanced crop cultivation NUE, the lower VNFs, and the healthy dietary patterns. Similarly, Han et al. (26) established scenarios on the future environmental footprints of healthy diets in China and found an age–gender-specific diet had the potential to reduce carbon, water, and ecological footprints toward 2100. Not consuming red meat can result in lower greenhouse gas emissions as well as reduce the mortality risk (27).

For the practical implementation of the integrated scenario, it is necessary to sketch the strategies to improve NUE of crop cultivation and to choose a religion-sensitive planetary health diet. To increase the crop cultivation NUE, knowledge about the 4R nutrient stewardship among farmers needs to be improved (i.e., right fertilizer source, right application rate, right application time, and right application place) (28). Selecting strategic genetically-improved crops has potential for a better NUE and a higher yield (29). Organic manure and biofertilizers which follow the principles of conservation agriculture (i.e., zero/minimum tillage, diversified crop rotation, and retention of crop residue) should be used in crop cultivation as this practice has been found to be effective for the soil and the environmental health protection (30). Nitrogen nanofertilizers as well as enhanced efficiency fertilizers increase crop cultivation NUEs by enhancing crop yield and reducing N losses to the environment (31). The efficient re-use of crop and animal waste through integrating crop and animal farms can also increase the NUE at the production level (32). To reduce the food N footprint by food consumption measures, the promotion of ethical and spiritual-based food behaviors among the religious communities is advised (3). Although it is important for individuals to follow a healthy diet, there is very little evidence on the religion-specific protein recommendation. We acknowledge that even with a framework of religion-sensitive food with altered protein supplies, changing dietary habits is challenging. Our recommended guideline for food intake is in line with the recommendations in the EAT–Lancet reference diet to double the consumption of healthy foods, at least halve the consumption of less healthy foods by 2050.

Besides increasing the NUE of crop cultivation and adopting a healthy diet to reduce the food N footprint, it is also essential that consumer-level food losses and food waste are reduced and that N-recycling is included in wastewater treatment. Efforts like sharing excess of food from religious festivities with those in need and converting food waste into bio-fertilizers are expected to be well-received globally as no religion supports the wasting of food (33). It has been estimated that around 8 Tg of religious waste, in the form of foods, flowers, and tree leaves, is thrown into the rivers in India as sacred offerings on an annual basis (34). It has also been noted that the N recovered from the wastewater treatment, and treated wastewater can be re-used providing it is of acceptable purity and has the original color, odor and taste, as required by religious strictures (3, 35).

### Limitations and Assumptions

There are several limitations to the religion-sensitive N-Calculator approach used in this study to estimate the food N footprint of the ISC by bringing partial adjustments to religious-based food behaviors. While the ISC consists of diverse religious communities, only four of them were considered in this study, and it was assumed that at least 75% of the population who identified with a religious faith strictly follow the dietary guidelines of that faith. In reality, however, individuals who identify with a certain religious group tend to be more flexible with regard to food choices. We prioritized the simplicity of this model and the estimates of the food N footprint of the ISC are approximate at best. While the adoption of EAT–Lancet reference diet would help to reduce the food N footprint of the ISC, this diet was designed for adults but not for children. Also, we did not consider the impact of unpredictable events, like the COVID-19 pandemic. The availability of more detailed data is required to achieve greater accuracy when calculating future N footprints.

## Conclusions

The overall strength of this study is that it realistically reveals the nature of religion-sensitive planetary health diets, and approaches to securing the nutrition required for the population in all religious communities with less N pollution. While the EAT–Lancet reference diet is designed to optimize human health and the environmental benefits globally, the gap between the protein consumption of this healthy diet and the actual diets of religious communities in the countries of the ISC is considerable. The religion-specific healthy diets with altered protein sources suggested in this study were designed to increase the intake of fruits, vegetables, “oil crops and pulses,” and other plant products, and reduce the consumption of cereals, starchy roots, and “milk and dairy products” to reducing the food N footprint of the ISC in the long run. In addition, the increase in the crop cultivation NUE approach recommended in this study will benefit the crop growers by reducing the Nr loss from crop production. The findings also have the potential to support the decision-makers responsible for formulating religion-sensitive policies for sustainable N management. The broader picture of the food N footprint presented for the ISC region can also contribute to the design of country-specific measures to reduce N losses in the agro–food system.

## Data Availability Statement

The raw data supporting the conclusions of this article will be made available by the authors, without undue reservation.

## Author Contributions

AD, KM, and AO: conceptualization and funding acquisition. AD and AO: methodology. AD: investigation, original draft preparation, visualization, and project administration. AO and KM: review and editing. KM: supervision. All authors contributed to the article and approved the submitted version.

## Funding

This research was funded by the Ministry of Education, Culture, Sports, Science and Technology (MEXT), Japan and JSPS KAKENHI, grant Nos. JP17H00794 and JP19K20496.

## Conflict of Interest

The authors declare that the research was conducted in the absence of any commercial or financial relationships that could be construed as a potential conflict of interest.

## Publisher's Note

All claims expressed in this article are solely those of the authors and do not necessarily represent those of their affiliated organizations, or those of the publisher, the editors and the reviewers. Any product that may be evaluated in this article, or claim that may be made by its manufacturer, is not guaranteed or endorsed by the publisher.
