# Supplementary material for: Food Nitrogen Footprint of the Indian Subcontinent Toward 2050
Source: Front Nutr. 2022 May 20;9:899431. doi: 10.3389/fnut.2022.899431 (PMC9165528; doi:10.3389/fnut.2022.899431)
Supplement: Supplementary file 1 [file Table_1.DOCX]

Supplementary Material

# Additional Sources of Data

The additional data sources used for calculation include fertilizer use by crop categories (1), N efficiency in animal production, slaughter and product collection efficiency, food and feed processing efficiency (2), feed composition and slaughter efficiency (3,4), protein content of food (5), N to protein conversion factors (6), calorie to protein conversion factors (7), livestock manure statistics (8), feed usage (2), agricultural residue usage (9), fisheries and aquaculture production (10), efficiency and recycling ratios of fish and seafood (11), food waste ratios (2,12), and protein intake facts (13).

# Supplementary Tables and Figures

## Supplementary Table

**Supplementary Table 1.** EAT-Lancet recommended planetary health diet

| Aggregate food categories | Calorie intake (kcal capita^-1^ day^-1^) | Protein intake (g capita^-1^ day^-1^) |
| --- | --- | --- |
| Cereals | 811 | 38·3 |
| Starchy roots | 39 | 1 |
| Oil crops and pulses | 450 | 2·7 |
| Vegetables | 78 | 0·6 |
| Fruits | 126 | 0·2 |
| Other plant products | 695 | 0·8 |
| Meat and offal | 92 | 1·6 |
| Milk and dairy products | 153 | 1·4 |
| Eggs | 19 | 0·4 |
| Fish and seafood | 40 | 3·9 |
| Total | 2503≈2500 | 50·9≈51 |

Source: Willett et al (2019), USDA (2021) (13,14).

## Supplementary Figures


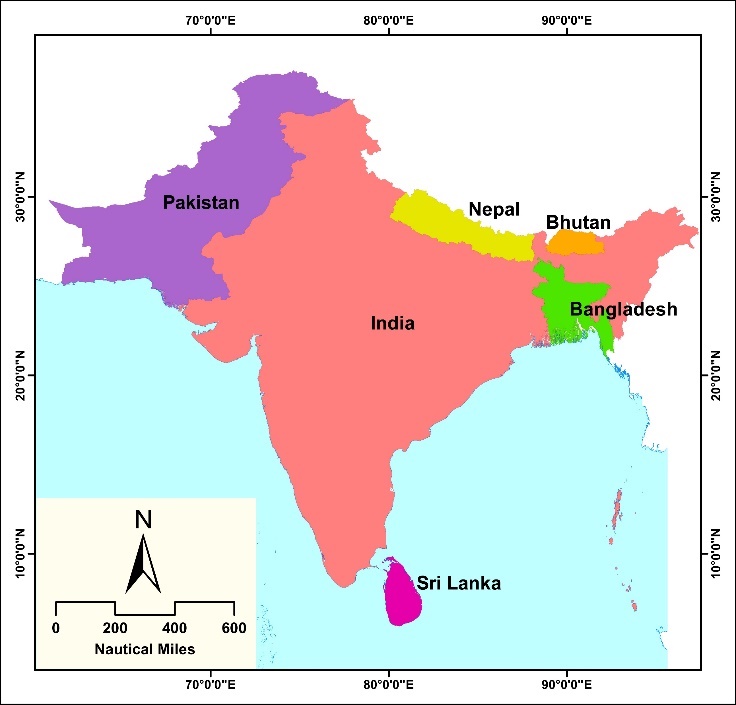


**Supplementary Figure 1.** Indian subcontinent

**Supplementary Figure 2.** Country-specific NUE in the ISC

| 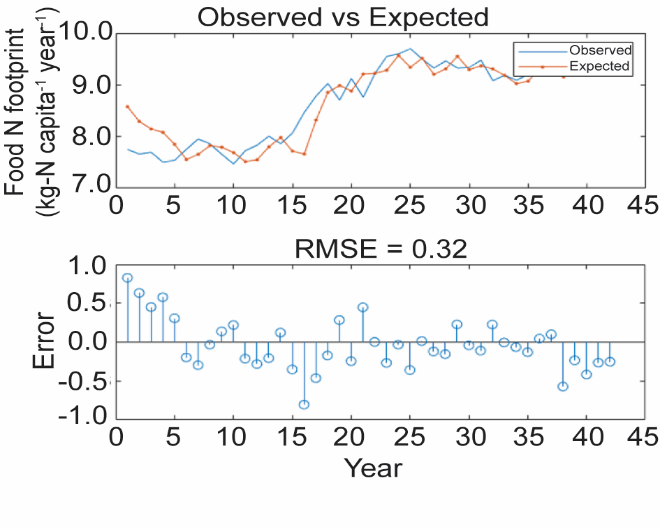 | 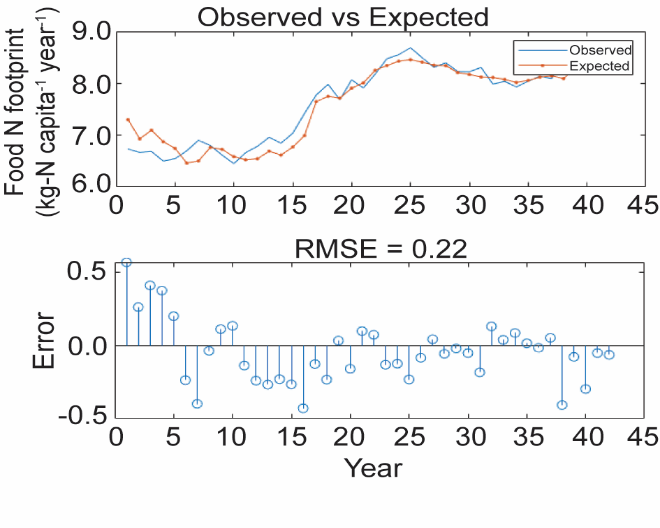 |
| --- | --- |
| (a) *BAU scenario* | (b) *NUE scenario* |
| 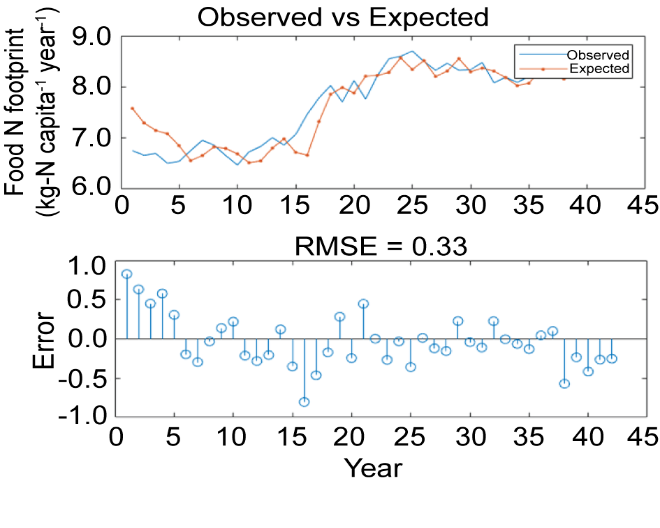 | 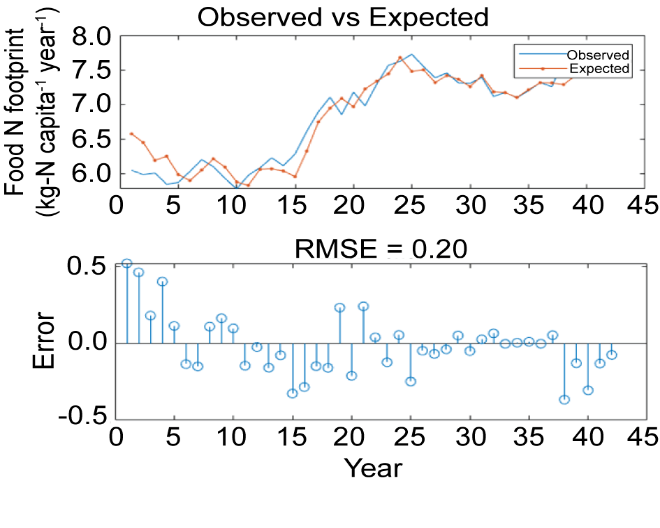 |
| (c) *EAT-Lancet scenario* | (d) *Integrated scenario* |

**Supplementary Figure 3.** RMSE values for LSTM-RNN model in different scenarios

# References

1. Heffer P, Gruère A, Roberts T. Assessment of fertilizer use by crop at the global level 2014-2014/15. (2017). https://www.fertilizer.org/images/Library_Downloads/2017_IFA_AgCom_17_134 rev_FUBC assessment 2014.pdf

2. Guo M, Chen X, Bai Z, Jiang R, Galloway JN, Leach AM, Cattaneo LR, Oenema O, Ma L, Zhang F. How China’s nitrogen footprint of food has changed from 1961 to 2010. *Environ Res Lett* (2017) **12**:104006. doi: 10.1088/1748-9326/aa81d9

3. MAFF. Document 1 of FY2008 joint meeting for improving feed self-sufficiency and production efficiency: Situation of feed (in Japanese). (2008). http://www.maff.go.jp/j/chikusan/souti/lin/l_siryo/koudo/h200422/pdf/data01.pdf

4. MAFF. Reference document of basic data for the 4th meeting of the Council of Food, Agriculture and Rural Area Policies in FY 2010 (in Japanese). (2010). http://www.maff.go.jp/j/council/seisaku/tikusan/bukai/h2204/pdf/data3_ref2_2.pdf

5. FAO. *Food balance sheets: A handbook*. Rome, Italy (2001). http://www.fao.org/3/x9892e/x9892e00.pdf

6. Mariotti F, Tomé D, Mirand PP. Converting nitrogen into protein - Beyond 6.25 and Jones’ factors. *Crit Rev Food Sci Nutr* (2008) **48**:177–184. doi: 10.1080/10408390701279749

7. CNF. Canadian Nutrient File. (2015) https://www.canada.ca/en/health-canada/services/food-nutrition/healthy-eating/nutrient-data/canadian-nutrient-file-2015-download-files.html

8. FAO. FAOSTAT Data. (2019) https://www.fao.org/faostat/en/#data/domains_table

9. Shibata H, Cattaneo LR, Leach AM, Galloway JN. First approach to the Japanese nitrogen footprint model to predict the loss of nitrogen to the environment. *Environ Res Lett* (2014) **9**:115013. doi: 10.1088/1748-9326/9/11/115013

10. FISHSTAT. Global fishery and aquaculture production statistics. (2019). http://www.fao.org/fishery/statistics/software/fishstatj/en

11. Oita A, Nagano I, Matsuda H. An improved methodology for calculating the nitrogen footprint of seafood. *Ecol Indic* (2016) **60**:1091–1103.

12. FAO. Global food losses and food waste – Extent, causes and prevention. Rome, Italy (2011). http://www.fao.org/3/mb060e/mb060e.pdf

13. USDA. The new nutrition facts label: Examples of different label formats. (2021). https://www.fda.gov/media/99203/download

14. Willett W, Rockström J, Loken B, Springmann M, Lang T, Vermeulen S, Garnett T, Tilman D, DeClerck F, Wood A, et al. Food in the Anthropocene: The EAT–Lancet Commission on healthy diets from sustainable food systems. *Lancet* (2019) **393**:447–492. doi: 10.1016/S0140-6736(18)31788-4
